# Nursing faculty perceptions of simulation culture readiness in Saudi universities: a cross-sectional study

**DOI:** 10.1186/s12912-023-01278-w

**Published:** 2023-04-07

**Authors:** Monir M. Almotairy, Maram Algabbashi, Sitah Alshutwi, Faygah Shibily, Fatmah Alsharif, Wedad Almutairi, Ahmed Nahari

**Affiliations:** 1 Nursing Administration and Education Department, College of Nursing, King Saud University, P.O Box: 642, Riyadh, 12372 Saudi Arabia; 2 Nursing Sciences and Research Department, College of Nursing, Umm Al-Qura University, Makkah, 24232 Saudi Arabia; 3 College of Nursing, King Saud bin Abdulaziz University for Health Sciences, Riyadh, 11481 Saudi Arabia; 4 King Abdullah International Medical Research Center (KAIMRC), Riyadh, 14611 Saudi Arabia; 5 Critical Care Nursing Department, King Abdulaziz University, Jeddah, 21589 Saudi Arabia; 6 Medical Surgical Nursing Department, King Abdulaziz University, Jeddah, 21589 Saudi Arabia; 7 Maternity and Pediatric Nursing Department, King Abdulaziz University, Jeddah, 21589 Saudi Arabia; 8 Nursing Medical Surgical Department, College of Nursing, King Saud University, Riyadh, 12372 Saudi Arabia

**Keywords:** Simulation Training, Readiness, Nursing Faculty, Baccalaureate nursing education, Saudi Arabia, COVID-19

## Abstract

**Background:**

Academic programs are increasing simulation-based learning in Saudi Arabia during COVID-19 pandemic; however, there is limited knowledge about these universities’ simulation culture readiness. Thus, the purpose of this study was to explore faculty perceptions of the readiness to integrate simulation into nursing programs.

**Methods:**

This cross-sectional correlational study recruited faculty members in four nursing colleges at Saudi universities using the simulation culture organizational readiness survey 36-item questionnaire. A total of 88 faculty members from four Saudi universities were included. Descriptive, Pearson’s correlation, independent sample t-test, and analysis of covariance analysis were utilized in this study.

**Results:**

Nearly 39.8% and 38.6% of the participants had *Moderately* and *Very Much* overall readiness for the simulation-based education (SBE), respectively. There were significant correlations between the *summary impression* on simulation culture readiness measures and simulation culture organizational readiness survey subscales (*p* < 0.001). Three simulation culture organizational readiness survey subscales (*defined need and suppor*t *for change*, *readiness for culture change*, and *time*, *personnel, and resource readiness*) and the *overall readiness for SBE* were correlated with age, years since highest degree, years of experience in academia, and years using simulation in teaching (*p* < 0.05). The *sustainability practices to embed culture* subscale and *summary impression* were only correlated significantly with the number of years using simulation in teaching (*p* = 0.016 and 0.022, respectively). Females had a significantly higher mean in the *sustainability practices to embed cultur*e subscale (*p* = 0.006) and the *overall readiness for simulation-based education* (*p* = 0.05). Furthermore, there were significant differences among the highest degree in the *overall readiness for SBE* (*p* = 0.026), *summary impression* (*p* = 0.001), the *defined need and support* subscale (*p* = 0.05), the *sustainability practices to embed culture* subscale (*p* = 0.029), and the *time, personnel, and resource readiness* subscale (*p* = 0.015).

**Conclusions:**

Favorable simulation culture readiness results suggest great opportunities to advance clinical competencies in academic curricula and optimize educational outcomes. Nurse academic leaders should identify needs and resources to enhance simulation readiness and encourage the integration of simulation in nursing education.

**Supplementary Information:**

The online version contains supplementary material available at 10.1186/s12912-023-01278-w.

## Background

In the last few decades, the use of simulation as an educational tool has grown significantly in nursing. Simulation-based education (SBE) has become an integral part of the nursing education programs worldwide. Challenges and issues related to clinical placements, patient safety, and ethical concerns have fostered the increased integration of simulation-based education into nursing curricula [1, 2]. Additionally, the COVID-19 pandemic has shown the importance and value of SBE as an educational tool, given the fact that COVID-19 pandemic has limited students’ direct experience with patient care and other valuable clinical opportunities [3, 4].

Simulation-based education is a useful pedagogical tool that enables nursing students to practice their clinical and decision-making skills [5]. A large and growing body of literature has shown that SBE enhances learning outcomes, improves decision-making skills, and helps nursing students to master nursing procedures and skills and recognize their learning needs independently [2, 6]. Evidence suggests that SBE helps to develop and improve students’ communication and collaboration skills [7]. Moreover, it has been reported that SBE promotes the self-confidence of nursing students and improves nursing students’ transition-to-practice [2, 6].

Despite the widely reported benefits of SBE, several challenges and barriers to implementation of SBE has been identified in the literature. The lack of trained simulation personnel has been identified as a factor that negatively impacts implementation of SBE [8]. Several efforts are reported to encourage faculty members to integrate simulation into their courses including providing simulation training through coursework, workshops, and webinars [9, 10]. Although the required human, infrastructural, and equipment resources are essential elements to the implementation of SBE, lack of organizational support and readiness to implementing the SBE has been identified as one of the most noted barriers [11]. The organizational support is an imperative factor for creating and enabling the environment needed for SBE integration into nursing education [12].

Organizational readiness for SBE is crucial for nursing education institutions to successfully utilize and integrate simulation into their academic and staff development programs [13]. The need to evaluate the organizational support and readiness for simulation has recently emerged in the nursing simulation literature [13, 14]. Evidence has shown that using a strategic change approach is highly linked to higher levels of commitment to simulation’s integration into nursing education. Therefore, the structured integration of SBE requires a clear strategic plan that involves organization leaders as stakeholders as well as educators who are delivering SBE [15]. Educators’ perceptions of readiness to integrate SBE into nursing education can play a crucial role in the integration process. The evidence suggests that educators’ perceptions towards SBE can enable or hinder the integration of SBE into nursing education [16–20]. Thus, exploring the faculty members’ perception of the readiness for SBE can foster the integration process into nursing curricula, which can facilitate the achievement of students’ learning competencies and outcomes.

At international levels, a large and growing body of literature has supported the integration of SBE into nursing curricula to enhance students’ learning outcomes [21, 22]. In Saudi Arabia, currently available evidence has shown that utilization of SBE has yield positive educational outcomes in terms of improving nursing students’ experiences, skills, and cognitive knowledge [23–25] and among health sciences students in Saudi Arabia [26, 27]. There have been several attempts and calls to foster the integration process in health sciences programs in Saudi Arabia [28, 29]. Qualitative evidence has shown that nursing faculty members in Saudi Arabia recognize and support the use of simulation to improve nursing students’ knowledge, skills, and attitudes [30]. Despite the fact that there is a great support for utilization of simulation in Saudi Arabia, little is known about faculty’s perception about readiness for integration of simulation into nursing undergraduate programs in Saudi Arabia. Evaluating faculty’s perception about simulation readiness can provide an insight from the frontline nurse academicians about current efforts on simulation integration in nursing education curricula. Thus, the purpose of this study is to (1) explore the faculty’s perception of about the simulation culture readiness at their academic institutions, and (2) examine the associations between the demographic characteristics and perception about simulation culture readiness among nursing faculty in Saudi Arabia.

### Theoretical framework

This study was guided by the organizational elements that shape simulation in nursing (OESSN) model [11]. This model evaluates the level of adopting and integrating simulation to enhance nursing, faculty, and institutional outcomes. According to the OESSN model, an institution with high simulation culture readiness prioritizes simulation in its philosophy and strategic objectives, defines needs and type of support to integrate simulation, allocates resources, prepares its employee to utilize simulation, and continuously monitors the proper integration process to achieve desired outcomes and maintain sustainable simulation integration. Thus, evaluating simulation culture readiness can provide an insight about the simulation integration progress at the institution to achieve desired outcomes at nursing, faculty, and institutional levels.

## Materials and methods

### Study design

This study utilized a cross-sectional correlational approach and was reported according to the STROBE checklist.

### Participants and setting

The study included faculty members from different universities at three cities in Saudi Arabia, utilizing a convenience sampling approach. The inclusion criteria were nurse faculty members working at study sites and able to read English language. Nurse faculty members who were not working for the study sites or not able to read and understand English language were excluded from the study. The approval was obtained from the study sites to collect data. The study investigators from these sites sent invitation emails of electronic questionnaires to a total of 176 nursing faculty members at the four universities. Data were collected between April 2022 and November 2022. A study sample size of at least 81 participants was targeted to provide a statistical power of ≥ 80% to detect a medium effect size of Cohen’s *f*^2^ = 0.15, assuming Type I error = 0.05 and planning for 5% missing data using G*Power software version 3.1.9.7.

### Instruments and Measures

The simulation culture organizational readiness survey (SCORS) was used in this study to evaluate the faculty’ perception of SBE integration into nursing curricula. The tool has been used by nurses, academic and clinical educators, and leaders developed to evaluate institutional and program readiness for integration of SBE [31]. It consists of 36 items measuring four areas (subscales) of readiness for integration of SBE, which are: (1) *defined need and support for change* [9 items], (2) *readiness for culture change* [11 items], (3) *time, personnel, and resource readiness* [12 items], and (4) *sustainable education development to embed culture* [4 items] (Leighton et al., 2018). The tool uses a five-point Likert scale (1 = not at all to 5 = very much), with a total score ranging from 36 to 180 indicating the *overall readiness for SBE* (0–36 = *not ready*, 37–72 = *a little*, 73–108 = *somewhat*, 109–144 = *moderately*, and 145–180 = *very much*.). The tool also includes two summative items (SCORS *summary impression)* to evaluate the current readiness and readiness six months prior, with scores ranging from 1 to 5 (1 = *not ready;* 2 = *getting ready*; 3 = *been ready, but not acting*; 4 = *ready to start to act*; 5 = *past ready and into action planning*) [31]. SCORS has established validity and reliability [31].

In the current study, two investigators reviewed SCORS items to ensure its readability and appropriateness to the context in Saudi universities. Moreover, the Cronbach’s alpha coefficients for *defined need and support for change*, *readiness for culture change*, time, *personnel, and resource readiness*, and *sustainable education development to embed* culture were 0.84, 0.87, 0.80 and 0.78, respectively. The Cronbach’s alpha coefficient for the whole scale was 0.89.

### Statistical analysis

Descriptive statistics were utilized for the participants’ demographic characteristics and SCORS four subscales, the *overall readiness for SBE*, and *summary impression*. Pearson correlation coefficient (*r*) was used to measure the strength and direction of associations between SCORS four subscales, the *overall readiness for SBE*, and *summary impression* and participants’ demographic characteristics (age, number of years since highest degree, number of years of experience in academia teaching, and number of years using simulation in teaching). Independent t-test was used to measure the strength and direction of association between SCORS four subscales, the *overall readiness for SBE*, and *summary impression* based on gender, being certified in simulation, and using simulation labs in teaching in the last 2 years. Analysis of Covariance (ANOVA) was used to measure the strength and direction of association between SCORS four subscales, the *overall readiness for SBE*, and *summary impression* based on the highest educational degree. A two-sided p-value < 0.05 was considered statistically significant. All analyses were performed using SPSS v27.0 (IBM Corp., Armonk, NY).

## Results

A total of 88 faculty members from four universities in Saudi Arabia were included in this study. Most participants were female (79.5%), Ph.D-prepared faculty (70.4%), had not obtained a simulation certification (92%), and used simulation in teaching in the last two years (65.9%). The participants’ age ranged from 26 to 65 years old (41.38 ± 7.68), years since the highest academic degree ranged from 0.5 to 36 years (8.64 ± 6.71), years of experience in academia ranged from 1 to 33 years (12.77 ± 8.29), and years using simulation in teaching ranged from 0 to 23 years (4.34 ± 4.12) (Table 1).

**Table 1:** The frequency and percentage regarding demographic data in study group (n = 88)

	N	%
**Age**		
< 35	14	15.9
35–40	30	34.1
40–50	23	26.1
> 50	21	23.9
Range	26–65	
Mean ± SD	41.384 ± 7.680	
**Gender**		
Female	70	79.5
Male	18	20.5
**Highest Degree**		
Bachelor (BSN)	4	4.5
Master (MSN)	22	25.0
Doctorate (PhD)	62	70.4
**Number of years since highest degree**		
< 5	30	34.1
5–10.	26	29.5
10–15.	10	11.4
> 15	22	25.0
Range	0.50–36	
Mean ± SD	8.6420 ± 6.712	
**Number of years of experience in academia teaching**		
< 5	13	14.8
5–10.	23	26.1
10–15.	18	20.5
> 15	34	38.6
Range	1–33.	
Mean ± SD	12.772 ± 8.286	
**Number of years using simulation in teaching**		
< 1	9	10.2
1–5.	47	53.4
5–10.	22	25.0
> 10	10	11.4
Range	0–23.	
Mean ± SD	4.3409 ± 4.124	
**Are you certified in simulation (e.g. CHSE, CHSOS)**		
No	81	92.0
Yes	7	8.0
**Have you used simulation labs in teaching in the last 2years**		
No	30	34.1
Yes	58	65.9

The scores for the *overall readiness for SBE* ranged from 69 to 178 (130.51 ± 27.28). Most of responses indicated favorable simulation culture readiness. Specifically, 2.3% of participants were in *a little* classification, 19.3% were in *somewhat*, 39.8% were in *moderately*, and 38.6% were in *very much* classifications (Table 2).

**Table 2:** Distribution of SCORS subscales and Overall Readiness for SBE in study group (n = 88)

	Not Ready		A Little		Somewhat		Moderately		Very Much		Scores	
	N	%	N	%	N	%	N	%	N	%	Range	Mean ± SD
**Defined Need and Support for Change**	0	0.0	4	4.5	5	5.7	35	39.8	44	50.0	14–45	34.761 ± 7.553
**Readiness for Culture Change**	0	0.0	3	3.4	15	17.0	33	37.5	37	42.1	18–55	40.022 ± 9.089
**Time, Personnel, and Resource Readiness**	0	0.0	5	5.7	20	22.7	35	39.8	28	31.8	16–58	40.704 ± 10.28
**Sustainability Practices to Embed Culture**	0	0.0	1	1.1	10	11.4	34	38.6	43	48.9	6–20.	15.022 ± 3.226
**Overall Readiness for simulation-based education**	0	0.0	2	2.3	17	19.3	35	39.8	34	38.6	69–178	130.51 ± 27.28
**Summary Impression**	2	2.3	4	4.5	17	19.3	28	31.8	37	42.0	2–20.	13.807 ± 4.065

Overall SCORS score = 0–36 (Not ready), 37–72 (A little), 73–108 (Somewhat), 109–144 (Moderately), and 145–180 (Very Much)

**Table 3:** The relationship between summary impression and SCORS’ subscales and Overall Readiness for SBE

	SUMMARY IMPRESSION	
	r	P-value
**Defined Need and Support for Change**	0.60	< 0.001*
**Readiness for Culture Change**	0.63	< 0.001*
**Time, Personnel, and Resource Readiness**	0.70	< 0.001*
**Sustainability Practices to Embed Culture**	0.58	< 0.001*
**Overall Readiness for simulation-based education**	0.71	< 0.001*

The study found significantly positive moderate correlations (ranging from 0.582 to 0.70) between the *summary impression* and SCORS subscales (*p* < 0.001) (Table 3). The study examined the correlations between the SCORS’s subscales and four demographic characteristics (age, years since highest degree, years of experience in academia, and years using simulation in teaching) (Table 4). Three SCORS subscales (*defined need and suppor*t *for change*, *readiness for culture change*, and *time*, *personnel, and resource readiness*) and the *overall readiness for SBE* were positively correlated with the four demographic characteristics (*p* < 0.05). However, the *sustainability practices to embed culture* subscale and *summary impression* were only correlated significantly with the number of years using simulation in teaching (*p* = 0.016 and 0.022, respectively).

**Table 4:** The relationship between SCORS’ subscales and demographic data (Age, years since highest degree, years of experience in academic teaching, and number of years using simulation in teaching)

	Age		Number of years since highest degree		Number of years of experience in academia teaching		Number of years using simulation in teaching	
	r	P-value	r	P-value	r	P-value	r	P-value
**Defined Need and Support for Change**	0.29	0.007*	0.24	0.027*	0.27	0.011*	0.30	0.005*
**Readiness for Culture Change**	0.27	0.010*	0.25	0.017*	0.36	0.001*	0.32	0.002*
**Time, Personnel, and Resource Readiness**	0.27	0.010*	0.24	0.025*	0.30	0.005*	0.33	0.001*
**Sustainability Practices to Embed Culture**	0.20	0.065	0.13	0.243	0.20	0.065	0.26	0.016*
**Overall Readiness for simulation-based education**	0.30	0.005*	0.26	0.017*	0.33	0.002*	0.35	0.001*
**Summary Impression**	0.09	0.385	0.01	0.961	0.07	0.529	0.24	0.022*

This study examined the differences between the SCORS subscales and *summary impression* among gender, highest degree, being certified in simulation, and simulation in teaching in the last two years (Tables 5, 6, 7 and 8). As shown in Table 5, females had a significantly higher mean in the *sustainability practices to embed cultur*e subscale (15.5 ± 3.3, *p* = 0.006) and the *overall readiness for SBE* (133.4 ± 29.1, *p* = 0.05). Furthermore, Table 6 showed that there were significant differences among the highest degree in the *overall readiness for SBE* (*p* = 0.026), *summary impression* (*p* = 0.001), and three SCORS subscales, which were the *defined need and support* subscale (*p* = 0.05), the *sustainability practices to embed culture* subscale (*p* = 0.029), and the *time, personnel, and resource readiness* subscale (*p* = 0.015). There were no significant associations for SCORS subscales, *overall readiness for SBE*, and *summary impression* based on the use of simulation in the last two years (Table 7) or being certified in simulation (Table 8).

**Table 5:** The relationship between SCORS’ subscales and gender

	Gender						T-test	
	Female			Male				
	Mean	±	SD	Mean	±	SD	T	P-value
**Defined Need and Support for Change**	35.40	**±**	7.77	32.28	**±**	6.21	1.58	0.118
**Readiness for Culture Change**	40.94	**±**	9.51	36.44	**±**	6.23	1.90	0.061
**Time, Personnel, and Resource Readiness**	41.54	**±**	10.96	37.44	**±**	6.26	1.52	0.132
**Sustainability Practices to Embed Culture**	15.50	**±**	3.35	13.17	**±**	1.79	2.85	0.006*
**Overall Readiness for simulation-based education**	133.39	**±**	29.09	119.33	**±**	14.42	1.98	0.05*
**Summary Impression**	14.21	**±**	4.24	12.22	**±**	2.90	1.88	0.063

**Table 6:** The relationship between SCORS’ subscales and highest degree

	Highest Degree									ANOVA	
	Bachelor (BSN)			Master (MSN)			Doctorate (PhD) or High				
	Mean	±	SD	Mean	±	SD	Mean	±	SD	F	P-value
**Defined Need and Support for Change**	31.25	**±**	5.74	31.86	**±**	8.32	36.02	**±**	7.11	3.043	0.05*
**Readiness for Culture Change**	39.50	**±**	5.26	36.82	**±**	10.18	41.19	**±**	8.70	1.928	0.152
**Time, Personnel, and Resource Readiness**	38.75	**±**	8.62	35.46	**±**	11.70	42.69	**±**	9.27	4.422	0.015*
**Sustainability Practices to Embed Culture**	15.50	**±**	1.29	13.46	**±**	3.56	15.55	**±**	3.04	3.677	0.029*
**Overall Readiness for simulation-based education**	125.0	**±**	16.39	117.59	**±**	30.58	135.45	**±**	25.29	3.793	0.026*
**Summary Impression**	13.25	**±**	1.71	11.091	**±**	4.43	14.81	**±**	3.60	7.905	0.001*

**Table 7:** The relationship between SCORS’ subscales and being certified in simulation

	Are you certified in simulation (e.g. CHSE, CHSOS)						T-test	
	No			Yes				
	Mean	±	SD	Mean	±	SD	T	P-value
**Defined Need and Support for Change**	34.72	**±**	7.71	35.29	**±**	5.88	-0.190	0.849
**Readiness for Culture Change**	40.19	**±**	9.19	38.14	**±**	8.15	0.568	0.571
**Time, Personnel, and Resource Readiness**	40.47	**±**	10.52	43.43	**±**	7.02	-0.729	0.468
**Sustainability Practices to Embed Culture**	15.15	**±**	3.29	13.57	**±**	2.07	1.244	0.217
**Overall Readiness for simulation-based education**	130.52	**±**	27.91	130.43	**±**	20.18	0.008	0.993
**Summary Impression**	13.69	**±**	4.16	15.14	**±**	2.67	-0.905	0.368

**Table 8:** The relation between SCORS’ subscales and using simulation in teaching

	Have you used simulation labs in teaching in the last 2years						T-test	
	No			Yes				
	Mean	±	SD	Mean	±	SD	T	P-value
**Defined Need and Support for Change**	33.57	**±**	7.91	35.38	**±**	7.36	-1.068	0.289
**Readiness for Culture Change**	39.17	**±**	7.49	40.47	**±**	9.85	-0.633	0.528
**Time, Personnel, and Resource Readiness**	38.60	**±**	9.58	41.79	**±**	10.54	-1.388	0.169
**Sustainability Practices to Embed Culture**	14.30	**±**	2.79	15.40	**±**	3.39	-1.522	0.132
**Overall Readiness for simulation-based education**	125.63	**±**	24.30	133.04	**±**	28.58	-1.209	0.230
**Summary Impression**	12.97	**±**	4.15	14.24	**±**	3.99	-1.402	0.164

## Discussion

This study explored the perceptions of faculty members at different academic institutions regarding the cultural readiness to integrate simulation into the nursing curriculums. Consistent with Moabi and Mtshali [12], our findings showed that at least 78% of participants moderately or strongly agreed that their academic institutions were ready for the simulation integration in academic curriculums, with the mean of the *overall readiness for SBE* at approximately 6.98 (SD ± 2.27). The most rated subscales were the *defined need and support for change* and *sustainability practice to embed culture*, indicating simulation acceptance as a tool to foster academic teaching. Our findings align with previous studies that showed a positive attitude and readiness toward simulation integration into curricula [32–35]. Our findings showed a higher impression of faculty toward the organizations’ readiness for SBE integration at the participation time (57.9%) than six months prior (47.7%), indicating the realization of SBE as an effective tool to overcome teaching barriers during the COVID-19 pandemic as supported in the previous studies [35, 36].

Our findings revealed that age, years since highest degree, years of experience in academia, and years of using simulation in teaching were significantly and positively correlated with the *overall readiness for SBE* and the three SCORS subscales (the *defined need and support for change*, *sustainability practice to embed culture*, and *time, personnel, and resource readiness)*. As these demographical characteristics are related, future studies can examine the association between these demographical factors have contributed to these significant findings. The remaining SCORS subscale (*sustainability practices to embed culture*) and *summary impression* were significantly correlated with solely years of using simulation in teaching. Females had a significantly higher mean score in the *overall readiness for SBE* and *the sustainability practices to embed culture* subscale. Having a doctorate degree had a significantly higher mean in three SCORS subscales (*defined need and support for change*, *sustainability practice to embed culture*, and *time, personnel, and resource readiness)* as well as in the *overall readiness for SBE* and *summary impression*. This finding is consistent with a relevant study in Saudi Arabia that identified higher degrees in nursing as an essential factor to integrate simulation in nursing curricula and provide rich learning experience [30]. Having a depth of knowledge and skills in research to evaluate simulation needs and current practices to sustain simulation-based education might explain this finding. However, future research should explore the influence of having a higher degree on simulation culture readiness. Although our findings are consistent with Moabi and Mtshali [12] in the agreement level (moderately to very much) with the participants’ academic institutions’ readiness for simulation integration in curricula and the distribution of age and gender groups in the sample of the study, most participants in their study had less than five years of experience in academia, whereas in our study the majority had more than or equal to ten years of experience. Thus, further research must evaluate the possible impact of age, gender, and years of experience on the readiness perception of simulation integration in curricula.

### Implications for nursing research, education, and practice

This study provides several crucial recommendations for research and practice. Possible factors that contribute to the variation in faculties’ perception of their institutions’ readiness for simulation integration as a teaching strategy in curricula in larger sample sizes must be explored. Advanced statistical modeling with a larger sample size may reveal a better understanding of facilitators and barriers to simulation integration in academia. Given the favorable readiness level shown in this study, further research can evaluate the implementation strategies of simulation across introductory and advanced courses in undergraduate and graduate nursing programs, the use of high-fidelity simulation technology, and evaluate its effectiveness in fostering learning outcomes. The academic practice can benefit from enhancing the integration of simulation throughout courses to foster learning. The findings of this study provide an insight for academic leaders to foster the integration of simulation-based learning into nursing curricula to facilitate the achievement of students’ learning competencies and outcomes. Furthermore, the integration of simulation-based learning into nursing curricula will prepare competent nurse graduates with required knowledge and skills to provide safe and high-quality nursing care across different clinical settings. Outcomes of simulation-based learning should be evaluated periodically to ensure the fulfillment of course competencies in meeting the demands of complex healthcare system.

### Limitations

The study had some limitations. The social desirability and self-reporting bias due to the study design might have led to over reported desired responses by participants. Additionally, the study had small sample size, which might limit the generalizability of the study findings. Furthermore, the study sample were from universities in three Saudi cities, so a large scale study on faculty members from Saudi universities in different cities and regions could provide a better insight and generalizable findings on the simulation culture readiness in Saudi universities.

## Conclusion

Simulation is a valuable tool to foster students’ learning and cognitive skills in prelicensure nursing programs by helping them determine the patients’ needs in the provided scenarios and prioritize nursing actions in a harm-free environment. Thus, simulation integration strategy in curricula can help equip prelicensure students with the knowledge and cognitive skills to handle complex health conditions across healthcare settings. In this study, faculty favorably rated their organizations’ readiness to integrate simulation in the curricula.

## Electronic supplementary material

Below is the link to the electronic supplementary material.

Supplementary Material 1

## Data Availability

The datasets used and/or analyzed during the current study available from the corresponding author on reasonable request.
