# Supplementary material for: Nursing faculty perceptions of simulation culture readiness in Saudi universities: a cross-sectional study
Source: BMC Nurs. 2023 Apr 7;22:105. doi: 10.1186/s12912-023-01278-w (PMC10079484; doi:10.1186/s12912-023-01278-w)
Supplement: Supplementary file 1 — Supplementary Material 1 [file 12912_2023_1278_MOESM1_ESM.docx]

Supplemental Table 1: Descriptive statistics for the Defined Need and Support for Change subscale

| **Items** | | | | **Defined Need and Support for Change** | | | | | | | | | | **% of agreement** | **Chi-square** | | |
| --- | --- | --- | --- | --- | --- | --- | --- | --- | --- | --- | --- | --- | --- | --- | --- | --- | --- |
|  |  |  |  | **None at All** | | **A Little** | | **Somewhat** | | **Moderately** | | **Very Much** | |  | **X^2^** | | **P-value** |
| **1** | **To what extent are innovation, experiential learning and quality student experiences clearly described as central to the mission and philosophy of your institution?** | **N** | 5 | | 1 | | 14 | | 27 | | 41 | | 82.27 | | 61.545 | 0.000 | |
|  |  | **%** | 5.7% | | 1.1% | | 15.9% | | 30.7% | | 46.6% | |  |  |  |  |  |
| **2** | **To what extent has your organization clearly defined the need to consider simulation-­‐based education (SBE) integration?** | **N** | 2 | | 3 | | 12 | | 24 | | 47 | | 85.23 | | 79.159 | 0.000 | |
|  |  | **%** | 2.3% | | 3.4% | | 13.6% | | 27.3% | | 53.4% | |  |  |  |  |  |
| **3** | **To what extent have administrators within your organization communicated a clear strategic vision for SBE?** | **N** | 3 | | 8 | | 19 | | 26 | | 32 | | 77.27 | | 33.250 | 0.000 | |
|  |  | **%** | 3.4% | | 9.1% | | 21.6% | | 29.5% | | 36.4% | |  |  |  |  |  |
| **4** | **To what extent have administrators within your organization provided a written commitment to SBE?** | **N** | 6 | | 9 | | 22 | | 23 | | 28 | | 73.18 | | 20.750 | 0.000 | |
|  |  | **%** | 6.8% | | 10.2% | | 25.0% | | 26.1% | | 31.8% | |  |  |  |  |  |
| **5** | **To what extent have administrators within your organization provided funding to support the commitment to SBE?** | **N** | 13 | | 16 | | 14 | | 27 | | 18 | | 64.77 | | 7.114 | 0.130 | |
|  |  | **%** | 14.8% | | 18.2% | | 15.9% | | 30.7% | | 20.5% | |  |  |  |  |  |
| **6** | **To what extent does your organization promote the need for SBE based on current evidence, standards, and guidelines?** | **N** | 1 | | 7 | | 19 | | 29 | | 32 | | 79.09 | | 41.318 | 0.000 | |
|  |  | **%** | 1.1% | | 8.0% | | 21.6% | | 33.0% | | 36.4% | |  |  |  |  |  |
| **7** | **To what extent is SBE currently being used as a teaching modality in your institution?** | **N** | 0 | | 6 | | 22 | | 39 | | 21 | | 77.05 | | 24.818 | 0.000 | |
|  |  | **%** | 0.0% | | 6.8% | | 25.0% | | 44.3% | | 23.9% | |  |  |  |  |  |
| **8** | **To what extent have the educators you work with articulated a need for SBE integration into the curriculum?** | **N** | 2 | | 7 | | 14 | | 35 | | 30 | | 79.09 | | 46.886 | 0.000 | |
|  |  | **%** | 2.3% | | 8.0% | | 15.9% | | 39.8% | | 34.1% | |  |  |  |  |  |
| **9** | **To what extent have the educators in your institution verbalized a commitment to SBE integration into the curriculum?** | **N** | 3 | | 5 | | 14 | | 45 | | 21 | | 77.27 | | 65.182 | 0.000 | |
|  |  | **%** | 3.4% | | 5.7% | | 15.9% | | 51.1% | | 23.9% | |  |  |  |  |  |

Supplemental Table 2: Descriptive statistics for the Readiness for Culture Change subscale

| **Items** | | | **Readiness for Culture Change** | | | | | **% of agreement** | **Chi-square** | |
| --- | --- | --- | --- | --- | --- | --- | --- | --- | --- | --- |
|  |  |  | **None at All** | **A Little** | **Somewhat** | **Moderately** | **Very Much** |  | **X^2^** | **P-value** |
| **10** | **To what extent is there a critical mass of professionals who already possess strong SBE:** | | | | | | | | | |
| **a** | **Knowledge** | **N** | 2 | 16 | 16 | 34 | 20 | 72.27 | 29.727 | 0.000 |
|  |  | **%** | 2.3% | 18.2% | 18.2% | 38.6% | 22.7% |  |  |  |
| **b** | **Skills** | **N** | 1 | 17 | 23 | 35 | 12 | 69.09 | 36.318 | 0.000 |
|  |  | **%** | 1.1% | 19.3% | 26.1% | 39.8% | 13.6% |  |  |  |
| **c** | **Positive Attitudes** | **N** | 1 | 11 | 16 | 34 | 26 | 76.59 | 37.568 | 0.000 |
|  |  | **%** | 1.1% | 12.5% | 18.2% | 38.6% | 29.5% |  |  |  |
| **11** | **To what extent do administrators support culture change including the efforts required to implement and sustain SBE program integration?** | **N** | 2 | 9 | 16 | 31 | 30 | 77.73 | 37.114 | 0.000 |
|  |  | **%** | 2.3% | 10.2% | 18.2% | 35.2% | 34.1% |  |  |  |
| **12** | **To what extent are there credentialed or trained simulationists who mentor/coach others, including, other simulationists?** | **N** | 13 | 19 | 10 | 32 | 14 | 63.41 | 17.114 | 0.002 |
|  |  | **%** | 14.8% | 21.6% | 11.4% | 36.4% | 15.9% |  |  |  |
| **13** | **To what extent does your organization have individuals who model SBE best practice?** | **N** | 8 | 16 | 16 | 35 | 13 | 66.59 | 23.932 | 0.000 |
|  |  | **%** | 9.1% | 18.2% | 18.2% | 39.8% | 14.8% |  |  |  |
| **14** | **To what extent are staff/faculty proficient in the use of technology? (I.e. computer systems, AV and IT systems)** | **N** | 2 | 5 | 11 | 38 | 32 | 81.14 | 60.750 | 0.000 |
|  |  | **%** | 2.3% | 5.7% | 12.5% | 43.2% | 36.4% |  |  |  |
| **15** | **To what extent are there graduate level prepared researchers available to assist in research to develop new knowledge, as appropriate to your organization’s mission?** | **N** | 6 | 10 | 17 | 32 | 23 | 72.73 | 24.386 | 0.000 |
|  |  | **%** | 6.8% | 11.4% | 19.3% | 36.4% | 26.1% |  |  |  |
| **16** | **To what extent are librarians available within your organization to help search for evidence-­‐based practice and related simulation resources?** | **N** | 17 | 10 | 10 | 32 | 19 | 65.91 | 18.477 | 0.001 |
|  |  | **%** | 19.3% | 11.4% | 11.4% | 36.4% | 21.6% |  |  |  |
| **17** | **To what extent are your librarians accessed to search for evidence-­‐ based practice and related simulation resources?** | **N** | 19 | 8 | 12 | 33 | 16 | 64.32 | 20.750 | 0.000 |
|  |  | **%** | 21.6% | 9.1% | 13.6% | 37.5% | 18.2% |  |  |  |
| **18** | **To what extent do you believe that now is the right time to implement a culture change to support SBE?** | **N** | 1 | 1 | 9 | 16 | 61 | 90.68 | 142.682 | 0.000 |
|  |  | **%** | 1.1% | 1.1% | 10.2% | 18.2% | 69.3% |  |  |  |

Supplemental Table 3: Descriptive statistics for the Time, Personnel, and Resource Readiness subscale

| **Items** | | | | **Time, Personnel, and Resource Readiness** | | | | | | | | | | **% of agreement** | | **Chi-square** | | | |
| --- | --- | --- | --- | --- | --- | --- | --- | --- | --- | --- | --- | --- | --- | --- | --- | --- | --- | --- | --- |
|  |  |  |  | **None at All** | | **A Little** | | **Somewhat** | | **Moderately** | | **Very Much** | |  |  | **X^2^** | | **P-value** | |
| **19** | **To what extent are fiscal resources available to support SBE in the following areas** | | | | | | | | | | | | | | | | | | |
| **a** | **Human resources (simulation personnel)?** | **N** | 8 | | 17 | | 20 | | 32 | | 11 | | 64.77 | | 19.841 | | 0.001 | |  |
|  |  | **%** | 9.1% | | 19.3% | | 22.7% | | 36.4% | | 12.5% | |  |  |  |  |  |  |  |
| **b** | **Education?** | **N** | 5 | | 14 | | 14 | | 39 | | 16 | | 70.68 | | 36.659 | | 0.000 | |  |
|  |  | **%** | 5.7% | | 15.9% | | 15.9% | | 44.3% | | 18.2% | |  |  |  |  |  |  |  |
| **c** | **Release time to lead integration of SBE?** | **N** | 8 | | 14 | | 19 | | 34 | | 13 | | 66.82 | | 22.568 | | 0.000 | |  |
|  |  | **%** | 9.1% | | 15.9% | | 21.6% | | 38.6% | | 14.8% | |  |  |  |  |  |  |  |
| **d** | **Development of physical learning spaces?** | **N** | 6 | | 11 | | 23 | | 35 | | 13 | | 68.64 | | 30.182 | | 0.000 | |  |
|  |  | **%** | 6.8% | | 12.5% | | 26.1% | | 39.8% | | 14.8% | |  |  |  |  |  |  |  |
| **e** | **Equipment?** | **N** | 4 | | 14 | | 20 | | 38 | | 12 | | 69.09 | | 37.000 | | 0.000 | |  |
|  |  | **%** | 4.5% | | 15.9% | | 22.7% | | 43.2% | | 13.6% | |  |  |  |  |  |  |  |
| **20** | **To what extent do employees in your institution have access to quality technology, including computers, audiovisual equipment, and other institutional technologies?** | **N** | 4 | | 4 | | 9 | | 45 | | 26 | | 79.32 | | 71.886 | | 0.000 | |  |
|  |  | **%** | 4.5% | | 4.5% | | 10.2% | | 51.1% | | 29.5% | |  |  |  |  |  |  |  |
| **21** | **To what extent is support available to learn and manage technologies that support education?** | **N** | 3 | | 7 | | 12 | | 44 | | 22 | | 77.05 | | 60.977 | | 0.000 | |  |
|  |  | **%** | 3.4% | | 8.0% | | 13.6% | | 50.0% | | 25.0% | |  |  |  |  |  |  |  |
| **22** | **To what extent are there existing simulation champions (people who will go the extra mile to advance simulation) in the current environment among:** | | | | | | | | | | | | | | | | | | |
| **a** | **Administrators?** | **N** | 8 | | 19 | | 19 | | 37 | | 5 | | 62.73 | | 35.864 | | 0.000 | |  |
|  |  | **%** | 9.1% | | 21.6% | | 21.6% | | 42.0% | | 5.7% | |  |  |  |  |  |  |  |
| **b** | **Clinicians?** | **N** | 6 | | 17 | | 26 | | 31 | | 8 | | 64.09 | | 27.114 | | 0.000 | |  |
|  |  | **%** | 6.8% | | 19.3% | | 29.5% | | 35.2% | | 9.1% | |  |  |  |  |  |  |  |
| **c** | **Educators?** | **N** | 3 | | 17 | | 21 | | 35 | | 12 | | 68.18 | | 31.773 | | 0.000 | |  |
|  |  | **%** | 3.4% | | 19.3% | | 23.9% | | 39.8% | | 13.6% | |  |  |  |  |  |  |  |
| **d** | **Technology Specialists?** | **N** | 12 | | 14 | | 25 | | 34 | | 3 | | 60.45 | | 33.023 | | 0.000 | |  |
|  |  | **%** | 13.6% | | 15.9% | | 28.4% | | 38.6% | | 3.4% | |  |  |  |  |  |  |  |
| **e** | **Administrative Assistants and Support Staff?** | **N** | 8 | | 21 | | 19 | | 33 | | 7 | | 62.27 | | 25.864 | | 0.000 | |  |
|  |  | **%** | 9.1% | | 23.9% | | 21.6% | | 37.5% | | 8.0% | |  |  |  |  |  |  |  |

Supplemental Table 4: Descriptive statistics for the Sustainability Practices to Embed Culture subscale

| **Items** | | | **Sustainability Practices to Embed Culture** | | | | | **% of agreement** | **Chi-square** | |
| --- | --- | --- | --- | --- | --- | --- | --- | --- | --- | --- |
|  |  |  | **None at All** | **A Little** | **Somewhat** | **Moderately** | **Very Much** |  | **X^2^** | **P-value** |
| **23** | **To what extent is the measurement and sharing of outcomes part of the culture of the organization in which you work?** | **N** | 1 | 10 | 19 | 35 | 23 | 75.68 | 37.909 | 0.000 |
|  |  | **%** | 1.1% | 11.4% | 21.6% | 39.8% | 26.1% |  |  |  |
| **24** | **To what extent are decisions regarding SBE influenced by:** | | | | | | | | | |
| **a** | **Clinicians?** | **N** | 2 | 12 | 24 | 35 | 15 | 71.14 | 35.523 | 0.000 |
|  |  | **%** | 2.3% | 13.6% | 27.3% | 39.8% | 17.0% |  |  |  |
| **b** | **Educators?** | **N** | 0 | 10 | 22 | 32 | 24 | 75.91 | 11.273 | 0.010 |
|  |  | **%** | 0.0% | 11.4% | 25.0% | 36.4% | 27.3% |  |  |  |
| **c** | **Administration?** | **N** | 1 | 8 | 23 | 24 | 32 | 77.73 | 36.659 | 0.000 |
|  |  | **%** | 1.1% | 9.1% | 26.1% | 27.3% | 36.4% |  |  |  |

Supplemental Table 5: Descriptive statistics for the Summary Impression

| **Items** | | | | | | | **SUMMARY IMPRESSION** | | | | | | | | | | **% of agreement** | | | **Chi-square** | | |
| --- | --- | --- | --- | --- | --- | --- | --- | --- | --- | --- | --- | --- | --- | --- | --- | --- | --- | --- | --- | --- | --- | --- |
|  |  |  |  |  |  |  | **Not Ready** | | **Getting Ready** | | **Been Ready But Not Acting** | | **Ready to Start to Act** | | **Past Ready & Into Action Planning** | |  |  |  | **X^2^** | **P-value** | |
| **25** | **Considering all of the SCORS indicator scores, how would you rate your organization's readiness for SBE integration?** | | | | **N** | | 3 | | 16 | | 18 | | 28 | | 23 | | 71.82 | | | 20.068 | 0.000 | |
|  |  |  |  |  | **%** | | 3.4% | | 18.2% | | 20.5% | | 31.8% | | 26.1% | |  |  |  |  |  |  |
| **26** | **Looking back 6 months, how would you have rated your organization’s readiness for SBE integration?** | | | | **N** | | 6 | | 23 | | 17 | | 28 | | 14 | | 64.77 | | | 16.205 | 0.003 | |
|  |  |  |  |  | **%** | | 6.8% | | 26.1% | | 19.3% | | 31.8% | | 15.9% | |  |  |  |  |  |  |
| **Plot your overall readiness level on the adjacent continuum.** | |  | **0** | **1** | | **2** | | **3** | | **4** | | **5** | | **6** | | **7** | | **8** | **9** | | | **10** |
|  |  | **N** | 1 | 2 | | 0 | | 3 | | 7 | | 9 | | 9 | | 15 | | 19 | 11 | | | 12 |
|  |  | **%** | 1.1 | 2.3 | | 0.0 | | 3.4 | | 8.0 | | 10.2 | | 10.2 | | 17.0 | | 21.6 | 12.5 | | | 13.6 |
|  |  | **Range (Mean±SD)** | | | | | | | | | | **0-10 (6.977±2.269)** | | | | | | | | | | |
